# SDR-Fi-Z: A Wireless Local Area Network-Fingerprinting-Based Indoor Positioning Method for E911 Vertical Accuracy Mandate

**DOI:** 10.3390/s25030823

**Published:** 2025-01-30

**Authors:** Rahul Mundlamuri, Devasena Inupakutika, David Akopian

**Affiliations:** Electrical and Computer Engineering Department, The University of Texas at San Antonio, San Antonio, TX 78249-0670, USA; devasena.prasad@gmail.com (D.I.); david.akopian@utsa.edu (D.A.)

**Keywords:** E911, 3D indoor localization, channel state information, floor detection, neural networks

## Abstract

The Enhanced 911 (E911) mandate of the Federal Communications Commission (FCC) drives the evolution of indoor three-dimensional (3D) location/positioning services for emergency calls. Many indoor localization systems exploit location-dependent wireless signaling signatures, often called fingerprints, and machine learning techniques for position estimation. In particular, received signal strength indicators (RSSIs) and Channel State Information (CSI) in Wireless Local Area Networks (WLANs or Wi-Fi) have gained popularity and have been addressed in the literature. While RSSI signatures are easy to collect, the fluctuation of wireless signals resulting from environmental uncertainties leads to considerable variations in RSSIs, which poses a challenge to accurate localization on a single floor, not to mention multi-floor or even three-dimensional (3D) indoor localization. Considering recent E911 mandate attention to vertical location accuracy, this study aimed to investigate CSI from Wi-Fi signals to produce baseline Z-axis location data, which has not been thoroughly addressed. To that end, we utilized CSI measurements and two representative machine learning methods, an artificial neural network (ANN) and convolutional neural network (CNN), to estimate both 3D and vertical-axis positioning feasibility to achieve E911 accuracy compliance.

## 1. Introduction

Location-based services and navigation technologies have played various roles in health, military, entertainment, and personal life. The Global Positioning System (GPS) is a broadly deployed navigation system based on the trilateration concept [1] and is primarily available and accurate outdoors. However, GPS positioning degrades or is denied in indoor environments due to significantly attenuated signal strengths and multipath effects [2]. Various alternative positioning systems have been proposed for GPS-denied environments [3]. In particular, indoor positioning systems (IPSs) based on terrestrial wireless signals are becoming increasingly popular to address public safety compliance requirements according to Enhanced 911 (E911) regulations for emergency response.

Most IPS methods exploit triangulation, trilateration, and fingerprinting-based methods. In particular, the trilateration and triangulation concepts [4,5] include the Angle of Arrival (AoA) [6,7,8], Time of Arrival (ToA) [9], and Time Difference of Arrival (TDoA) [10,11] methods and their variations. These methods need a direct line of sight between the multiple reference beacons and the user, along with the locations of these beacons. In fingerprinting-based methods, positioning algorithms use received location-dependent signal patterns called signatures or fingerprints. Typically, signal fingerprint maps are created through offline surveying by associating fingerprints with the locations on an area grid. Then, real-time positioning matches received wireless fingerprints with the pre-surveyed map entries to infer the likely location. As Wireless Local Area Networks (WLANs or Wi-Fi) are broadly deployed in indoor infrastructures, IPS methods often use Wi-Fi signaling fingerprints collected from access points (APs). Despite an overhead effort in offline fingerprint map creation, the fingerprinting technique is advantageous. It exploits signal variations due to multiple paths, it can work even with a single AP, and the locations of APs are not required. Even in non-line-of-sight environments, Wi-Fi-based fingerprinting methods enable indoor positioning [12].

Federal Communication Commission (FCC) E911 regulations have evolved and currently mandate additional vertical accuracy guidelines to support emergency responders in indoor multi-story building environments, helping identify floor levels. Earlier solutions using cellular signals, such as [11,13], did not comply with mandated accuracies. More advanced IPS methods adopted built-in sensors [14,15] for improved accuracy. In particular, the hybrid approaches [16,17] used barometer sensors to achieve compliance with the FCC vertical accuracy requirements. In addition to barometer sensors, the hybrid approach in [17] exploits Wi-Fi measurements as well.

While less accurate Wi-Fi algorithms for vertical positioning have been reported in the past [18,19,20], the recent evolution of Wi-Fi positioning algorithms using CSI fingerprints provides essential accuracy gains and could comply with the FCC requirements. This work presents a feasibility study on exploiting a CSI-based E911-compliant solution trained using machine learning without barometric sensors. The CSI is collected using software-defined radio (SDR).

This paper introduces a novel 3D indoor positioning approach that integrates CSI fingerprint recognition with advanced machine learning techniques, including artificial neural networks (ANNs) and convolutional neural networks (CNNs). Unlike traditional Received Signal Strength Indicator (RSSI)-based methods, which suffer significant performance degradation when the number of access points (APs) is limited, the proposed method achieves a high accuracy with a single AP. By leveraging the fine-grained, multipath-sensitive nature of CSI, this approach ensures reliable and precise positioning even in challenging environments with minimal infrastructure. The methodology eliminates the need for barometric sensors or other specialized hardware, addressing issues like sensor calibration and drift while reducing computational overhead. The experimental results demonstrate the effectiveness of the approach, with a vertical mean absolute error (MAE) of 0.80 m for 90% of locations and a floor detection accuracy of 94.62%, significantly outperforming established methods such as NextNav and Polaris Wireless.

In addition to its hardware efficiency, this paper highlights the robustness of CSI against environmental challenges, such as signal interference and multipath effects, which commonly degrade the performance of RSSI-based methods. The integration of machine learning models further enhances this robustness, enabling the extraction of unique location-specific features from CSI subcarrier measurements for precise positioning. The paper clarifies the key contributions of this approach, including its ability to meet and exceed FCC’s E911 vertical accuracy mandates in GPS-denied environments, providing a cost-effective and scalable solution. By addressing the limitations of existing methods and discussing the impact of limited AP availability, this work demonstrates the potential of CSI combined with machine learning to advance the state-of-the-art in indoor localization and emergency response technologies.

The remainder of this paper is structured as follows: Section 2 provides detailed background on E911 vertical positioning requirements. Then, Section 3 describes the CSI-based method. Section 4 summarizes experimental results and studies a competitive fingerprinting approach addressing these requirements. Finally, Section 5 provides concluding remarks.

## 2. E911 Z-Axis Positioning

While technological advances have improved the ability of emergency responders to locate callers, challenges remain, particularly in locating them in multi-story buildings [21]. In multi-story environments, firefighters, medics, and police officers frequently fail to locate them unless the caller provides specific details, like a floor number, room number, suite number, or any other important information about the area. This makes it challenging to identify and support the caller in their emergency.

The FCC has been rolling out E911 in phases. The vertical aspect of the caller’s location, the Z-axis, is essential to identify the caller in emergencies quickly. The Z-axis is typically expressed as height above the ground floor number and was not provided with most E911 services. The current E911 phase requires that network carriers incorporate the Z-axis metric. The FCC mandates Z-axis accuracy within three meters for 80% of indoor emergency calls. Service providers are expected to meet this prerequisite in the top 50% of the US markets by April 2023 [22].

Most carrier services use several standardized E911-compliant technologies to provide the user’s location when emergency calls are made. For example, if available, GPS satellite signals, network assistance, and hybrid solutions support the accuracy of 50 to 150 m. The accuracy would be affected if the calls are placed particularly from indoors due to the low received signal strength and multipath effects inside multi-story constructions or high-rise buildings [2,23].

Initial approaches for 3D indoor localization in GPS-denied environments were based on cellular-based signals. For fingerprinting-based approaches, the RSS of the cellular signals are collected from the base stations, and the collected data are used to build a fingerprinting database [11,13]. However, due to the shadowing effect, not all cellular transmissions from multiple stations penetrate deep into structures, resulting in incomplete fingerprints and blind spots [12,14]. Then, various sensors are explored to refine 3D indoor localization, including accelerometers, barometers, gyroscopes, and magnetometers, which are currently built-in in wireless devices. In particular, barometer outputs enhance the Z-axis accuracy. Kalman filters are typically employed to process the results of the sensors for location estimation [14,15].

Recently, NextNav and Polaris Wireless demonstrated positioning solutions meeting FCC requirements on E911 Z-axis accuracy [4]. NextNav uses barometric sensors already available in wireless devices. The readings of the sensors are sent to NextNav cloud, and the device data are compared to local conditions taken from NextNav altitude stations, generating a Z-axis location measurement or altitude information [16]. The company exploits local weather stations tracking atmospheric pressure readings across thousands of cities and towns. With access to a precise reading close to a calling party’s location, NextNav translates a device’s reported pressure reading into an accurate height calculation above the earth’s curve. For X-Y-axis coordinates, NextNav uses a proprietary hybrid method incorporating assisted GPS and provides the estimated location to the emergency service. NextNav also deployed terrestrial signaling infrastructure for GPS-denied scenarios.

Polaris Wireless employs a proprietary hybrid software-based solution that integrates location measurements from cellular signals and Wi-Fi, such as signal strengths, assisted built-in GPS receivers, timing advance and time of arrival measurements, and barometers, among others. Enhanced Cell-ID (ECID) and Wi-Fi data are used to generate a hybrid Z-axis location estimate [17].

Barometric sensors are critical in the current state-of-the-art Z-axis positioning [24]. One should notice, though, that not all wireless devices include these sensors. In addition, the calibration of these sensors may impact the accuracy of the techniques. In addition, sensors have drift and bias errors that should be compensated for, adding a computational burden on mobile devices [25].

## 3. Wi-Fi CSI-Based Localization

This section presents an approach that addresses E911 Z-axis requirements without barometric sensors. As mentioned, Wi-Fi-based indoor location methods gained popularity because of their wide deployment and accessibility. Wi-Fi fingerprinting contains two phases, offline surveying, and online positioning. In the offline phase, received signal measurements from APs are collected at a grid of surveying locations called reference points (RPs) hereafter. A group of RPs with associated signal measurements form a fingerprint map for the surveyed area.

The wireless measurements are noisy and distorted by multipath effects. For this reason, many samples of surveyed measurements are collected for each location and used to train machine learning models to optimally associate measurement samples with the closest RP locations. This study used two representative neural network (NN) algorithms [26] for machine learning.

In the online stage, after a mobile device collects online measurements, the measurement data are compared with the fingerprint map, which associates offline surveyed measurements with the grid of RP location. The comparison is based on machine learning approaches and identifies the most likely RP or RPs associated with the online measurement.

Early Wi-Fi fingerprinting approaches relied on received signal strength indicator (RSSI) measurements commonly collected using network interface cards [27]. Quite accurate RSSI-based horizontal location estimations have been reported in the literature, such as a median error of 1.48 m [28]. More accurate measurements using SDR receivers may have a slightly better accuracy, such as 1.2 m of median error and 1.49 m of mean error [29]. However, RSSI-based methods suffer from the quality of measurements due to multipath interference and non-line-of-sight indoor environments. Their location accuracy heavily relies on the number of available APs, with significantly degraded performance for three or fewer APs. Therefore, lately, Wi-Fi-based CSI fingerprinting methods have gained popularity for indoor localization [30]. CSI-based methods provide the ability to benefit from the multipath effects, which are location-specific. The Wi-Fi 802.11 a/g/n/ac/ax standards [31] employ orthogonal frequency division multiplexing (OFDM). In OFDM, the data are divided into multiple concurrent subcarriers (SCs) where each SC is orthogonal to another, and each SC is modulated with digitally modulated techniques. The channel impacts each subcarrier by applying different signal loss multipliers. The set of these SC multipliers is called CSI, which is used as a signal location-dependent fingerprint. The CSI fingerprint samples are surveyed during the offline phase for each location to create the radio map, and they are used to train the machine learning models for the online operation phase by associating the observed CSI fingerprints with the closest entries of the radio map and infer the location.

To assess E911 compliance for estimating vertical Z-axis location, this study used SDR technology to collect CSI measurements and machine learning using the conventional artificial neural network (ANN) and convolutional neural network (CNN) for matching radio map entries with the online observed measurements.

### 3.1. Experiment Methodology

**Figure 1:**
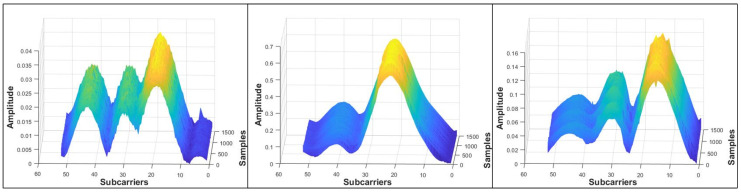
Density plot of RPs 2, 35, and 68 from three different floors.

In our experiments, the CSI measurements were collected utilizing the fast-prototyping WLAN OFDM-based SDR receiver [29]. CSI is a sub-carrier-level channel information. Figure 1 shows the plots of CSI measurements for all SCs on three RP locations. RP2, RP35, and RP68 belong to the ground, first, and second floors, respectively. Repetitive 1500 samples were collected for each RP to distinguish the pattern of the samples. One can see that at each RP, all the samples exhibit similar patterns, whereas, with a different location, the CSI pattern is unique.

**Figure 2:**
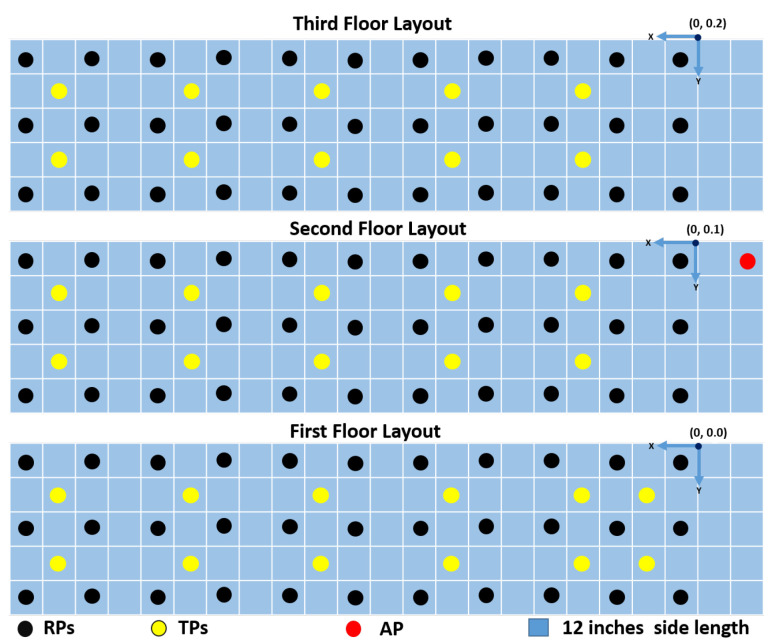
Hallway area layout of a three-story building.

The data collection site was a representative office construction: Biotechnology Sciences and Engineering Building at the University of Texas at San Antonio. A three-story fragment of the building was selected. For each floor, we surveyed 33 RPs, totaling 99 RPs, with a granularity of 60.96 cm (2 ft) between RPs. We chose 32 testing points (TPs) in-between RPs for validation: 12 TPs on the first floor, and 10 TPs each for the second and third floors. The hallway depicted a cluttered environment with desks, chairs, and lockers. Figure 2 shows the indoor hallway layout with a single AP. The total volume was around 1152 $m^{3}$ (3780 ${\mathrm{ft}}^{3}$), and the sides were around 6.5 m × 1.5 m × 10.97 m (21 ft × 5 ft × 36 ft). We describe a sample as a successful reception of a beacon frame at the receiver, offering RSS measurements and 52 measurements of CSI. For each RP and TP, we collected 1500 and 500 samples, respectively. We used a NI-USRP front-end along with an ASUS ROG Laptop, ASUS Tek Computer Inc., Taipei, Taiwan. (quad-core Intel i7-6700HQ processor, 32 GB RAM, and Windows 10) and the proposed SDR-Fi. The SDR took around 3 min to collect 1500 samples and 1 min to collect 500 samples. A total of 148,500 samples were collected for RPs and 16,000 samples for TPs.

### 3.2. Applied Neural Networks

#### 3.2.1. Artificial Neural Network

The collected surveyed CSI data are passed as input to the multi-layer ANN [32]. The input layer size is, thus, dependent on the CSI dimensions, which is 52; the output layer size corresponds to the number of locations. The ANN was trained to classify input CSI into target radio-map locations. The input dimensions of the ANN for the one-dimensional (1D) CSI data correspond to the sub-carriers (per sample, per location), [52 × 1]. Initially, the inputs are computed and passed as weights and biases assigned to neurons of the first hidden layer. The outputs of each layer and the weighted sums for the network are, thus, calculated. The ANN was characterized by selecting hyper-parameters through a randomized search [33]. The gradient was minimized based on a chosen mean square error loss function. The architecture of the ANN in this study consists of three hidden layers with 300, 150, and 100 neurons per layer, respectively. The chosen activation function is the hyperbolic tangent sigmoid (tansig) for all the hidden layers. This function calculates a layer’s outputs from its net inputs and returns the value of each net input’s element between −1 and 1. The overall training of the ANN employed a multilayer backpropagation training algorithm, called scaled conjugate gradient (SCG). SCG provides a tradeoff between accuracy and fast convergence. The adjustable model parameters (weights and biases) are updated at each iteration, and the network randomly divides the data into training, validation, and testing subsets. As the output layer implements a Softmax function to map the output weights to the estimated locations, each output neuron corresponds to an RP, and the predicted probabilities for each location are the final weights.

#### 3.2.2. Convolutional Neural Network

The architecture of a 1D CNN [32] for location estimation with a Z-axis component is illustrated in Table 1. The 1D signal consisting of CSI magnitudes is the model input. The CNN model extracts location signatures using convolutional layers. The architecture additionally constitutes a pooling layer followed by one or more fully connected layers for performing the location classification. The last fully connected layer always consists of the same number of neurons as the number of RPs.

The hyper-parameters that characterize the CNN were chosen through a randomized search [33]. The 1D CNN had one convolutional layer with 16 filters and a kernel size of [1 × 50], followed by a Rectified Linear Unit (ReLU) as the activation layer, and Batch Normalization. The Batch Normalization converts the inputs to have a zero mean or unit variance. Then, we have a cross-channel normalization with a window channel size of 3. Both the aforementioned normalizations stabilize the neural network by preventing vanishing gradients and overfitting [34,35] and improve performance. Finally, the fully connected Softmax layers give the final probabilities for every RP location. The earlier layers of the CNN’s output are flattened into a single vector, each representing the possibility that a particular feature is a location label. As for the network optimization, we used the Stochastic Gradient Descent with Momentum (SGDM) [35,36] algorithm to minimize the loss and adjust the weights (model parameters) and learning rates (hyper-parameters).

## 4. Experimental Results and Validation

This section presents the performance metrics used in the experimental validation, followed by testing configurations and a comparative analysis of the results. In this work, we evaluate the performance of the indoor localization in terms of both the X-Y-Z and Z-axis positions on a 3D coordinate system.

### 4.1. Performance Metrics and Testing Configuration

In the online phase, each TP sample is evaluated against the trained model (on RPs). We use mean absolute error (MAE) as one of the performance metrics by using the weights of each output class (based on the RPs) per TP evaluation, thus computing a 3D centroid location, i.e., $\left({\hat{x}}_{TP,k},{\hat{y}}_{TP,k},{\hat{z}}_{TP,k}\right)$ [37]. Furthermore, we subtract the coordinates from the known TP coordinates used in the said evaluation, which are denoted by $\left(x_{TP,k},y_{TP,k},z_{TP,k}\right)$, respectively. The locations are then compared against the true TP locations by evaluating K TPs to compute the MAE ($\epsilon_{xyz}$) as given in (1). For computing the vertical Z-axis error, we use MAE ($\epsilon_{z}$) as a performance metric to evaluate the vertical positioning performance as in (2). For the final location, we estimate the z-centroid location (${\hat{z}}_{TP}$) using the probability-weighted centroid method.

(1) $$\begin{array}{cc} \epsilon_{xyz} & =\frac{1}{K}\sum_{k=1}^{K}\sqrt{\left({\hat{x}}_{TP,k}-x_{TP,k}\right)+\left({\hat{y}}_{TP,k}-y_{TP,k}\right)+\left({\hat{z}}_{TP,k}-z_{TP,k}\right)} \end{array}$$

(2) $$\begin{array}{cc} \epsilon_{z} & =\frac{1}{K}\sum_{k=1}^{K}|\left({\hat{z}}_{TP,k}-z_{TP,k}\right)| \end{array}$$

For 3D indoor localization, the two customized neural networks, ANN and CNN, were used to evaluate CSI-based fingerprinting. For the ANN model, 70% of the samples were used for training, 20% for validation, and 10% for testing. We used a total of three hidden layers with a size of 300/150/100 neurons. For each layer, we used tangent sigmoid as an activation function. A Softmax transfer function was used in the output layer because it is a multi-location classification problem. We used mean square error as the loss function and SCG back-propagation as a training function. For training, the 1D CNN model used a kernel size of [1×50] with 16 filters. The samples were divided into 90% for training and 10% for validation. The learning rate was set at 0.001. We trained the 1D CNN model using SGDM, with a momentum η of 0.9 and a weight decay factor for L2 Regularization of 0.0005. For testing both models, we utilized the samples collected at the TPs on the three floors in the experimental environment.

### 4.2. Results

For testing 3D indoor localization, we selected 32 TPs, with 500 samples at each TP (see Figure 2). We chose 12 TPs for the first floor and 10 TPs each for both the second and third floors. Thus, we had 6000 samples for the first floor and 5000 samples each for the remaining two floors (Figure 2).

#### 4.2.1. Vertical Z-Axis Error

**Figure 3:**
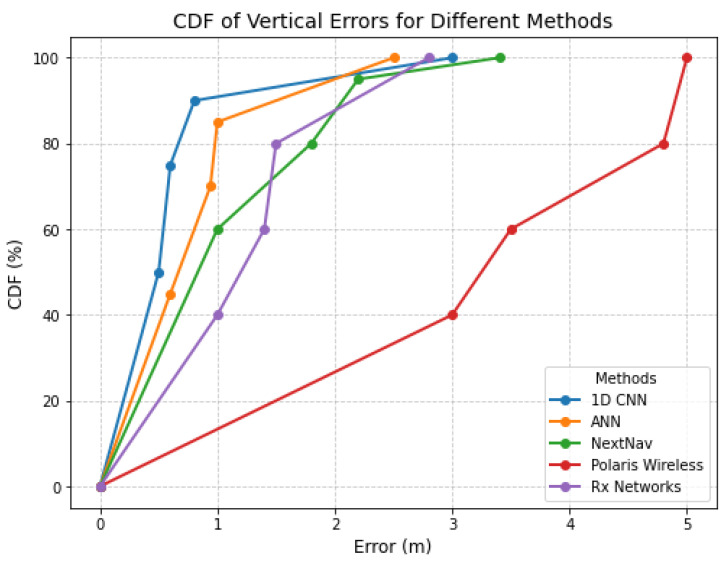
Vertical error comparison with existing methods.

This subsection compares the vertical or Z-axis error with the proposed neural network models (ID CNN and ANN) to the reported MAEs of barometric sensor-based NextNav, HLE-based Polaris Wireless, and assisted GPS-based Rx Networks. Which can been in the CDF Plot(Figure 3). We used our most optimized configurations for the 1D CNN and ANN (Section 3.1). We used 52 SCs as feature inputs, 1500 training samples per RP, and 500 testing samples per TP. Table 2 shows the results for the Z-axis error in indoor environment. The best performance is observed for the CNN method with a 0.80 m mean absolute error for 90% of locations while the ANN achieves a 0.94 m mean absolute error for 75% of the locations. Both models in this study outperformed NextNav, Polaris Wireless, and Rx Networks, which reported Z-axis errors of 1.8 m at 80%, 4.8 m at 80%, and 1.5 m at 80%, respectively.

#### 4.2.2. 3D XYZ-Axis Error and Floor Detection

In this subsection, we compare the total 3D error with the proposed neural network models, CNN and ANN. We can see that both the 1D CNN and ANN exhibit comparable performance for X-Y-Z-axis MAE (Table 2). The MAE of the CSI-based CNN is 0.9973 m, and the standard deviation is 0.9527 m, whereas, for ANN, it is 1.0753 m and 0.66 m for the mean and standard deviation, respectively. Additionally, we observe an improvement in the standard deviation due to a more acceptable resolution in the CSI. In other words, the CSI translates to overall precision improvements because the network trains with an enhanced channel estimate.

**Figure 4:**
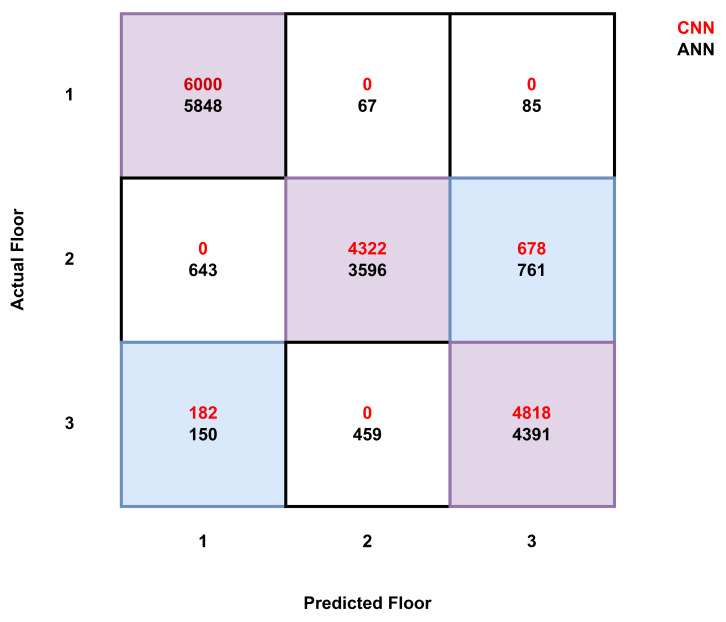
Confusion matrix for floor detection with CNN and ANN models.

Figure 4 shows the confusion matrix of 16,000 testing samples floor classification for both the CNN and ANN models. Out of 16,000 testing samples, the CNN correctly classified 15,140 samples and the ANN 13,835 sample, which yielded 94.62% and 86.46% floor classification accuracies for the CNN and ANN, respectively. Overall, a stable performance is observed in a cluttered indoor environment (multi-story building) for Z- and X-Y-Z-axes estimations with unique location signatures in the fine-grained CSI measurements.

The results demonstrate the potential use of CSI-based fingerprinting for accurate vertical positioning without barometers to comply with the E911 requirements. As our experiments were conducted in a supervised environment, the results may vary in different indoor structures. Nevertheless, the testing environment was a representative office building, and the results demonstrate competitive outcomes.

#### 4.2.3. Comprehensive Evaluation of Model’s Performance

Table 3 presents a detailed comparative analysis of the proposed ANN and CNN models for floor detection, evaluated using precision, recall, and F1-score metrics. Both models exhibited a strong classification performance, with the CNN generally outperforming the ANN across the majority of metrics. For Floor 1, the CNN achieved a slightly higher precision (0.967) and F1-score (0.983) compared to the ANN (0.964 precision and 0.975 F1-score), indicating the CNN’s superior ability to minimize false positives while maintaining a high classification accuracy. This demonstrates the robustness of both models in accurately identifying Floor 1 instances.

For Floors 2 and 3, the CNN consistently demonstrated a superior performance, achieving a precision of 1.000 and an F1-score of 0.927 for Floor 2, compared to the ANN’s precision of 0.865 and F1-score of 0.811. This suggests the CNN’s enhanced capability to correctly classify instances while reducing false positives. Similarly, for Floor 3, the CNN outperformed the ANN in precision (0.867 vs. 0.843) and achieved a higher F1-score (0.929 vs. 0.872). However, the ANN achieved a higher recall for Floor 3 (0.903 compared to the CNN’s 0.867), indicating its greater ability to capture true positives for this floor.

The macro-average metrics provide an aggregate view of the models’ performance across all floors. The CNN achieved a superior macro-average precision (0.945) and F1-score (0.946) compared to the ANN (0.891 precision and 0.886 F1-score). Moreover, the CNN’s macro-average recall (0.955) surpassed that of the ANN (0.884), reinforcing its robustness in achieving a high classification accuracy across multiple floors. These findings establish the CNN as the more reliable model for floor detection in indoor positioning systems. While the ANN demonstrated a competitive performance, particularly in terms of recall for Floor 3, the CNN’s overall consistency and higher scores across key metrics make it the preferred choice for scenarios demanding high precision and a balanced performance.

#### 4.2.4. Challenges and Limitations

This paper highlights the potential of CSI-based 3D positioning methods but also acknowledges certain challenges and shortcomings that warrant further discussion. One key limitation is the reliance on an offline fingerprinting phase, which requires significant manual effort to collect CSI measurements at multiple reference points across the area of interest. This process can be time-consuming, particularly in large or dynamically changing indoor environments where fingerprints may need frequent updates due to environmental changes. Additionally, while the proposed method demonstrates robust performance using a single access point (AP), the accuracy may degrade in environments with extreme signal interference or physical barriers that significantly alter CSI patterns. These factors can introduce noise or inconsistencies in the training and testing phases of the neural networks, potentially affecting performance.

Another challenge lies in the scalability of the approach when applied to real-world scenarios with diverse indoor structures and layouts. The experimental results were obtained in a controlled environment, and performance may vary in highly cluttered or irregular indoor spaces. Furthermore, while the approach eliminates the need for additional hardware like barometric sensors, it still requires specialized SDR equipment for CSI measurements, which may not be readily accessible in all deployments. Lastly, the computational requirements of training advanced machine learning models, particularly CNNs, can pose a challenge in resource-constrained settings, though this can be mitigated by leveraging cloud-based processing. Addressing these challenges in future work, such as automating fingerprint collection and improving model adaptability to diverse environments, will be critical for the broader adoption and practical deployment of CSI-based 3D positioning systems.

## 5. Conclusions

The FCC’s vertical location accuracy metric is 3 m above or below the handset for 80% of all wireless E911 calls. This standard applies to all handsets that have the capability to support floor detection, regardless of technology. The industry has demonstrated feasibility to meet these expectations using hybrid approaches integrating wireless radio frequency measurements and barometric sensors. This paper demonstrates that the CSI-based fingerprinting approach achieves a better accuracy without barometric sensors when using CSI measurements from even one Wi-Fi access-point beacon. The overhead of the proposed approach is in the offline surveying of areas of interest. Multi-story building operators may be interested in deploying the alternative CSI-based technology for more accurate location guidance of E911 responders to service their tenants in need. The CSI-based positioning results are demonstrated using conventional machine learning methods employing ANNs and CNNs. The results can be even improved more when using more advanced ML techniques. The CNN model demonstrated an accuracy of 0.80 m of vertical error and 0.99 of 3D error, whereas our ANN model demonstrated an accuracy of 0.94 m of vertical error and 1.07 m of 3D error. A floor detection rate above 90% was obtained with CSI. The experimental testbed, thus, validates the feasibility of CSI for floor detection in indoor multi-story buildings.

Future work will focus on addressing the identified challenges to enhance the practicality and scalability of the proposed CSI-based 3D positioning system. Automating the offline fingerprinting process through techniques like robotic surveying or crowdsourced data collection could significantly reduce the labor-intensive nature of creating fingerprint maps. Additionally, developing adaptive models that can update dynamically in response to environmental changes, such as furniture rearrangement or signal interference, will improve the system’s robustness in real-world scenarios. Research into lightweight, edge-compatible versions of the neural networks could make the approach more feasible for resource-constrained environments, enabling real-time processing on standard Wi-Fi devices. Furthermore, extending the experimental validation to diverse indoor settings, such as malls, hospitals, and high-rise buildings, will ensure the method’s generalizability. Finally, exploring hybrid models that integrate CSI with other data sources, such as accelerometers and LiDAR, could further enhance the accuracy and reliability of 3D indoor positioning while maintaining compliance with E911 mandates.

## Figures and Tables

**Table 1:** 1D Convolutional Neural Network Architecture Overview.

Layer	Configuration
Conv1D	Kernel Size: [1×50] and Filters: 16
ReLU Activation	-
Batch Normalization	-
Cross Channel Normalization	Window Channel Size: 3
Fully Connected	Number of training locations: 99 neurons
Softmax	Probability outputs

**Table 2:** Comparative 3D positioning performance results of proposed ANN and CNN models.

Models		XYZ Error			Vertical Error	
		Mean (m)	Std. dev (m)		Mean (m)	Floor Detection Accuracy (%)
CNN		0.9973	0.9527		0.8042	94.62
ANN		1.0753	0.6600		0.9407	86.46

**Table 3:** Comparative performance results of proposed ANN and CNN models for floor detection.

Floor/Metric	CNN Metrics			ANN Metrics		
	Precision	Recall	F1-Score	Precision	Recall	F1-Score
Floor 1	0.967	1.000	0.983	0.964	0.986	0.975
Floor 2	1.000	0.864	0.927	0.865	0.763	0.811
Floor 3	0.867	1.000	0.929	0.843	0.903	0.872
Macro Avg Precision	0.945	-	-	0.891	-	-
Macro Avg Recall	-	0.955	-	-	0.884	-
Macro Avg F1-Score	-	-	0.946	-	-	0.886

## Data Availability

The data supporting the reported results in this study are not publicly available due to privacy and ethical restrictions. However, additional details and datasets may be made available upon reasonable request to the corresponding author.
